# Multicentre Trial Evaluating the Safety and Tolerability of Estetrol-Drospirenone Combined Oral Contraceptive in Postmenarchal Female Adolescents

**DOI:** 10.3390/jcm14248832

**Published:** 2025-12-13

**Authors:** Angelica Lindén Hirschberg, Lali Pkhaladze, Kristina Gemzell-Danielsson, Kai Haldre, Kateryna Ruban, Nina Flerin, Guillaume Chatel, Dan Apter

**Affiliations:** 1 Department of Women’s and Children’s Health, Karolinska Institutet, 17177 Stockholm, Sweden; angelica.hirschberg.linden@ki.se (A.L.H.); kristina.gemzell@ki.se (K.G.-D.); 2 Department of Gynecology and Reproductive Medicine, Karolinska University Hospital, 17176 Stockholm, Sweden; 3 Prof. Zhordania and Prof. Khomasuridze Institute of Reproductology, Tbilisi 0186, Georgia; lpkhaladze@yahoo.com; 4 East Tallinn Central Hospital Women’s Clinic, 10138 Tallinn, Estonia; kai.haldre@itk.ee; 5 Estetra SRL, a Wholly Owned Subsidiary of Gedeon Richter Plc., 4000 Liège, Belgium; cmaxbiotech@gmail.com (K.R.); flerinni@gedeonrichter.com (N.F.); 6 Department of Obstetrics and Gynaecology, University of Helsinki, 00100 Helsinki, Finland; dan.apter@vlmedi.fi

**Keywords:** adolescents, estetrol/drospirenone, safety, tolerability, cycle control, dysmenorrhea

## Abstract

**Objectives**: This study aims to evaluate the safety and tolerability of estetrol/drospirenone in adolescents. **Methods**: In this Phase 3 open-label study, postmenarchal adolescents (12–17 years) received estetrol (E4)/drospirenone (DRSP) 15 mg/3 mg orally for six cycles (24 active/4 placebo regimen). Safety was evaluated through adverse event (AE) reporting. Participants also recorded daily pill intake, bleeding/spotting, dysmenorrhea, and pain medication use in e-diaries. Descriptive statistics were used. **Results**: Of 112 enrolled participants, 105 received treatment (mean age: 15.2 years), and 84.8% were completers. No serious treatment-related AEs or safety concerns were observed. Nausea and dysmenorrhea (each 1.9%) were the most common treatment-related AEs. Over 71% of participants took all tablets in each cycle. The percentage of participants with unscheduled bleeding and/or spotting decreased from 45.8% (Cycle 1) to 14.5% (Cycle 5), and the number of days with unscheduled bleeding and/or spotting decreased from nine to six days per cycle. The scheduled bleeding and/or spotting rate ranged between 77.4% and 90.5%, with a duration decreasing from six to four days in Cycle 1 to Cycle 5. Absence of scheduled bleeding increased from 9.5% in Cycle 3 to 22.6% in Cycle 5. The proportion of participants reporting dysmenorrhea decreased by 34.8%, with a median visual analogue scale score dropping from 5.0 at baseline to 3.7 at Cycle 6. Pain medication use decreased from 63.9% to 31.6% in Cycle 6. **Conclusions**: The use of E4/DRSP in adolescents raised no safety concerns, was well tolerated, resulted in a clear and stable cyclic bleeding pattern, and reduced pain associated with dysmenorrhea.

## 1. Introduction

Education and contraception both play important roles in preventing unintended pregnancy in adolescents [1]. However, adolescents have the lowest level of contraceptive knowledge and use [1] and about 11% of births worldwide are given by mothers aged between 15 and 19 years [2]. Pregnancy in adolescents may result in multiple health complications [2,3]. Hormonal contraception is prescribed frequently to adolescents for a variety of medical conditions beyond birth control, such as irregular or heavy menstrual bleeding, dysmenorrhea, hirsutism, or acne [4,5,6,7]. A U.S. study found that 33% of adolescent pill users (15–19 years) use it solely for these non-contraceptive reasons, while 67% use it for dual purposes [8].

Over the last decades, the benefit-risk profile of combined oral contraceptives (COCs) has improved due to new estrogen/progestogen combinations and lower doses of estrogens [9]. At the same time, these new options have not been fully evaluated in users younger than 18 years old [6]. Estetrol (E4) 15 mg (as monohydrate, equivalent to 14.2 mg estetrol)/drospirenone (DRSP) 3 mg is the only combined oral contraceptive containing a native estrogen with a unique selective mode of action, suggestive of a potentially better safety profile. This includes a low impact on haemostasis parameters and the potential to reduce the risk of venous thromboembolism associated with COC use [10,11]. Two parallel, multi-national phase 3 clinical studies assessing the safety and efficacy of estetrol/drospirenone 15 mg/3 mg, one conducted in the U.S./Canada and one in Europe/Russia, demonstrated a high contraceptive efficacy, a predictable bleeding profile, and a favourable safety and tolerability profile in users ≥ 16 years of age [12,13]. Estetrol/drospirenone was generally well-tolerated, with a discontinuation rate due to treatment-related adverse events reaching 8%, including only 2.8% related to bleeding complaints [14].

We conducted a six-month phase 3 study with estetrol/drospirenone in healthy postmenarchal adolescents, with the primary objective of assessing the safety of estetrol/drospirenone in this population [15]. We evaluated tolerability by recording bleeding patterns and the occurrence of dysmenorrhea [15].

## 2. Materials and Methods

This multicentre, phase 3, open-label, single-arm study was conducted from December 2020 to November 2023 in six European countries (Estonia, Finland, Georgia, Latvia, Poland, and Sweden). Investigators conducted the study in accordance with the Declaration of Helsinki and Good Clinical Practices, and under the Paediatric Investigation Plan (PIP) agreed upon with the European Medicines Agency (EMA). Local Ethics Committees at the study centres approved the study, and study participants, regardless of age, provided written informed assent or consent before study entry. Depending on local requirements, the legal representative(s) also provided written informed consent.

Investigators enrolled postmenarchal adolescents, requesting a COC for contraceptive or therapeutic use, aged 12 to 17 years and two months of age (inclusive) and a menstrual cycle length of 21–45 days. The eligibility criteria for contraceptive use were based on those outlined in the product label [16]. We excluded participants with any condition representing contraindications or precautions for use of COCs, undiagnosed abnormal vaginal bleeding within the past six months, any clinically relevant infection, diseases or disorders, (history of) malignancy, use of an injectable contraceptive method, an intrauterine device, a hormonal subdermal contraceptive implant or using medication that could lead to interactions with COCs [16].

After screening, eligible participants entered a pre-treatment cycle and kept a daily e-diary to record absence/occurrence of vaginal bleeding/spotting, dysmenorrhea using a visual analogue scale (VAS) [10-point scale; 0 = no pain, 10 = worst pain imaginable]), and the use of pain medication. Participants who were already using a COC stopped this method at the end of their regular cycle and entered the pre-treatment cycle without using a COC. After the pre-treatment cycle, participants started their six-month estetrol/drospirenone 15 mg/3 mg oral treatment on the first day of menstruation. Participants continued recording absence/occurrence of vaginal bleeding/spotting, dysmenorrhea, the use of pain medication for dysmenorrhea and recorded estetrol/drospirenone intake in their e-diary. Study staff scheduled follow-up visits at the end of Cycles 3 and 6, as well as at the end of treatment (EoT). At each visit, study staff asked participants about adverse events (AEs), measured vital signs, performed urine pregnancy tests, and dispensed study medication. Adherence recorded in the e-diary was verified by comparing electronic entries with returned tablet counts at each visit. At screening and study exit, investigators also performed a physical examination and 12-lead electrocardiograms (ECG), and collected blood for haematology, serum chemistry and pregnancy testing. The study aimed to include ≥100 participants, a number considered adequate to evaluate the safety of a new COC in adolescents and aligned with the requirements of the EMA-approved PIP.

We evaluated safety based on data from participants who received one or more doses of the study medication. Primary safety parameters included the type and frequency of AEs occurring during the treatment period. Investigators assessed the severity of AEs and their relatedness to the study drug. We summarised AEs by system organ class and preferred terms using the Medical Dictionary for Regulatory Activities (MedDRA) version 24.1. For the evaluation of bleeding pattern and dysmenorrhea parameters, we included all participants who received at least one dose of the study medication and had at least one post-baseline assessment of a secondary endpoint. If a subject had more than two consecutive days of missing bleeding data in the e-diary, that specific cycle was excluded from the analysis.

We analysed scheduled bleeding/spotting, unscheduled bleeding/spotting, and absence of scheduled bleeding/spotting data by cycle according to the methods suggested by Mishell et al. [17] adapted for a 24/4-day regimen. Cycle 6 bleeding data were not collected because the last 3 days of the scheduled bleeding period fell outside of the 6-month trial period. For the evaluation of dysmenorrhea, we used the individual cycle average of the three highest VAS scores (10-point scale; 0 = no pain, 10 = worst pain imaginable) in the pre-treatment Cycle (baseline) and Cycles 1, 3, and 6, and at the last evaluable cycle. This method was chosen to capture clinically meaningful peak pain intensity most likely to impact daily functioning. We conducted a subgroup analysis on participants with an average VAS baseline score above the median for the whole group.

Operational definitions for scheduled and unscheduled bleeding, evaluable-cycle criteria, and procedures for handling missing data are provided in Appendix A and Appendix B.

All data were summarised using descriptive statistics (number and percentage for categorical variables and mean, standard deviation, median, minimum, and maximum for continuous variables) using SAS^®^ software, version 9.4. No inferential statistical testing was planned or performed, in accordance with the predefined statistical analysis plan.

## 3. Results

**Figure 1:**
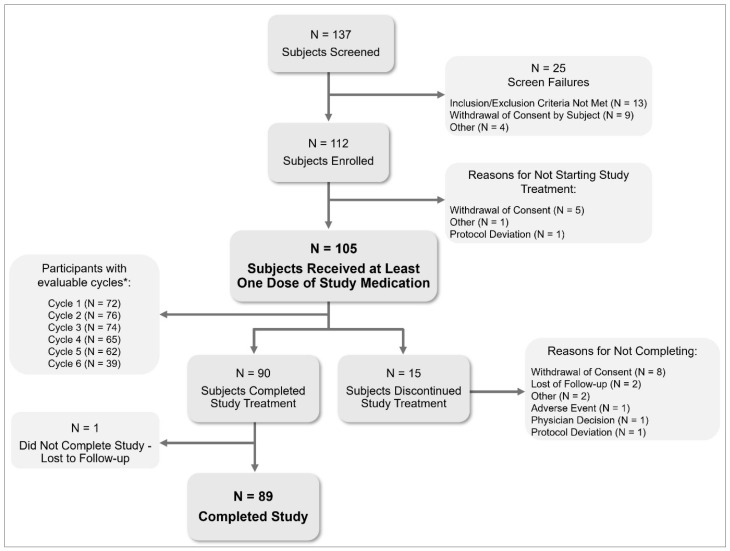
Subject disposition in a six-month paediatric study with estetrol/drospirenone (N = 105). * Evaluable cycles: cycles with a length of 22–35 days or with ≥2 hormone-free days at the end of the cycle. Subjects can have multiple reasons for screen failure.

Investigators screened 137 and enrolled 112 participants in the pre-treatment cycle, of whom 105 started estetrol/drospirenone treatment (Figure 1). Eighty-nine (84.8%) completed the study. One participant was lost to follow-up after completing the study treatment, and 15 participants (14.3%) discontinued the study treatment. Participants had a mean (SD) age of 15.2 (1.2) years, with the majority (79.0%) aged between 15 and 17 years. Most participants (91.4%) were starters (not using hormonal contraception within two months before screening), and 83.8% reported a history of dysmenorrhea (Table 1).

In each cycle, over 71% of participants took all tablets (active and placebo). The percentage of participants who took all active tablets increased from 77.8% in Cycle 1 to 94.9% in Cycle 6.

### 3.1. Safety

Fifty-four participants (51.4%) reported a total of 113 Treatment-Emergent AEs (TEAEs; Table 2). Most TEAEs were of mild intensity (75.2%). Four events (3.5%) reported by two participants were of severe intensity: dysmenorrhea, abdominal pain, and two events of depressed mood (reported by both participants). One participant discontinued due to TEAEs. This was a 16-year-old starter who experienced dysmenorrhea and heavy menstrual bleeding and had a history of dysmenorrhea.

Twelve participants (11.4%) reported 15 treatment-related TAEs (13.3% of 113 TEAEs). The most common were nausea and dysmenorrhea (Table 2). There were no serious TEAEs reported, and no clinically significant changes were observed in vital signs, ECG, or physical exams. We observed a slight mean weight increase of 0.38 kg (SD ± 3.3 kg) and body mass index (SD ± 0.38 kg/m^2^), from baseline to EoT (Table 2). No pregnancies were reported, but the number of exposed cycles was not recorded. No psychiatric adverse events or clinically relevant laboratory abnormalities were observed.

### 3.2. Cycle Control

**Figure 2:**
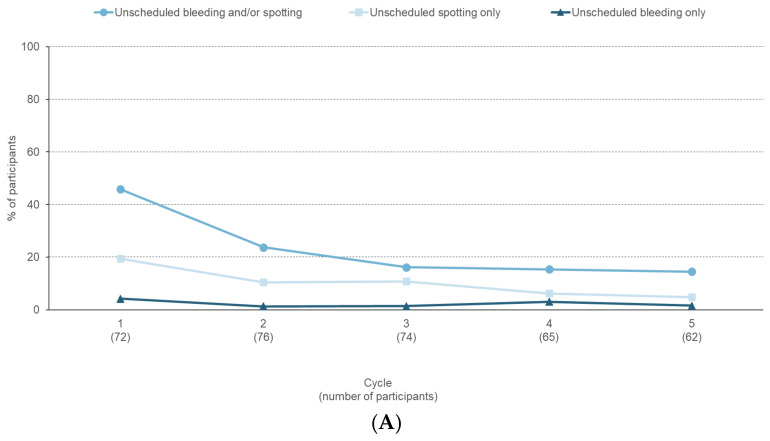

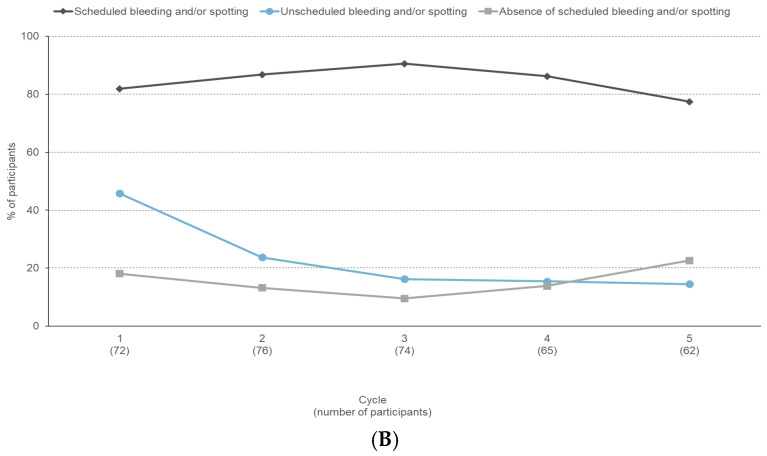
Bleeding profile reported during a six-month paediatric study with estetrol/drospirenone (N = 105). (**A**) Percentage of participants with unscheduled bleeding/spotting episodes, (**B**) Percentage of participants with unscheduled bleeding/spotting, scheduled bleeding/spotting, and absence of scheduled bleeding/spotting.

The proportion of participants reporting unscheduled bleeding and/or spotting decreased from 45.8% in Cycle 1 to 14.5% in Cycle 5 (Figure 2A). In participants reporting unscheduled bleeding and/or spotting, the median duration decreased from nine days in Cycle 1 to six days in Cycle 5. The proportion of participants reporting scheduled bleeding and/or spotting varied between 77.4% and 90.5% (Figure 2B). In participants reporting scheduled bleeding and/or spotting, the median duration decreased from six days in Cycle 1 to four days in Cycle 5. Absence of scheduled bleeding increased from 9.5% (Cycle 3) to 22.6% (Cycle 5) (Figure 2B).

**Figure A1:**
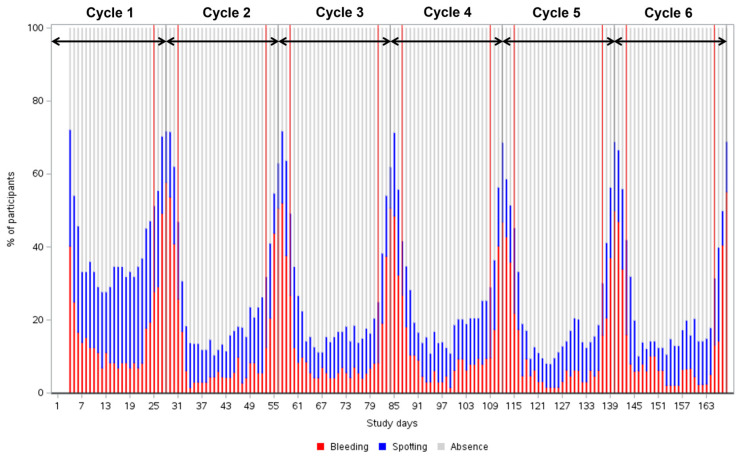
Percentage of participants with any type of vaginal bleeding and/or spotting over time reported during a six -month pediatric study with estetrol/drospirenone (N = 105). Arrows delineate pill cycles (from Day 1 to Day 28). Red lines delineate the scheduled bleeding period that occurs between Day 25 and Day 3 of the next cycle. Cycle 1: n = 72; Cycle 2: n = 76 for Cycle 2; Cycle 3: n = 74; Cycle 4: n = 65; Cycle 5: n = 62; Cycle 6: n = 39.

Over the six treatment cycles, a clear and stabilising cyclic bleeding pattern emerged, with the number of bleeding and spotting days decreasing after Cycle 1 (Appendix C Figure A1).

### 3.3. Dysmenorrhea

During the pre-treatment Cycle 63.9% participants used medication for relief of dysmenorrhea. Pain medication use decreased to 56.3%, 38.2% and 31.6% in cycles 1, 3, and 6, respectively (Appendix D Table A3). Mean (SD) number of days per cycle with use of pain medication decreased from 2.1 (5.0) days at baseline to 0.6 (1.0) days at Cycle 6.

**Figure 3:**
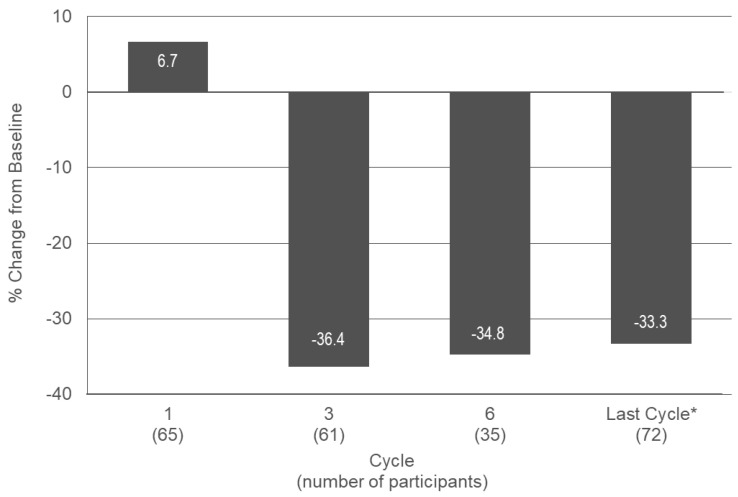
Median change from baseline in VAS score for dysmenorrhea in treatment cycles 1, 3, and 6 and at the last cycle during a six-month paediatric study with estetrol/drospirenone (N = 105). * Last observation carried forward. VAS: visual analogue scale. Baseline: n = 83; Median (min, max) VAS score: 5.0 (0.0, 9.7).

The median VAS score for dysmenorrhea was 5.0 at baseline and in Cycle 1, and decreased to 3.0, 3.7, and 3.5 in Cycles 3, 6, and EoT, respectively, representing decreases from baseline of 36.4%, 34.8%, and 33.3% (Figure 3, Appendix E Table A4).

In the subgroup of participants with more severe dysmenorrhea at baseline (0 = no pain, 10 = worst pain imaginable), the median VAS score was 6.7 at baseline and Cycle 1. It decreased to 3.7, 4.3, and 4.0 in Cycles 3, 6, and EoT, respectively. This represents a decrease from baseline of 44.5%, 42.3% and 41.7%, at cycles 3, 6, and EoT, respectively, indicating a more pronounced effect compared to the total group of participants.

## 4. Discussion

No safety concerns were observed with the use of estetrol/drospirenone over six cycles in adolescents, and only one participant (1.0%) discontinued due to AEs. Most reported AEs were changes in menstrual bleeding, headache, breast pain, abdominal pain and nausea, which is consistent with the safety profile of estetrol/drospirenone in the adult population as observed in the phase 3 studies [14]. A comparison between the younger age group (16–25 years, n = 1736) and the older age group (26–35 years, n = 1491) in the estetrol/drospirenone phase 3 studies showed no difference in incidence of AEs [18]. Remarkably, no treatment-related headaches, a common AE associated with COC use [19], were reported in this adolescent study. During the six-month treatment period, the mean body weight increased by 0.38 kg, which is within the expected weight gain over six months for adolescents in the age range studied [20].

A pooled analysis of previous phase 3 studies showed that estetrol/drospirenone is highly effective, with a Pearl Index of 1.52 in women aged 16–35 years and 1.61 in those aged 16–25 years [21]. The long half-life of DRSP (30 h [22]) and estetrol (24–28 h [23,24]) may enhance efficacy by reducing the impact of missed pills, which is of particular importance for adolescents, who tend to have lower treatment adherence [25]. Phase 3 estetrol/drospirenone studies analysed the relationship between adherence and pregnancy and found that the likelihood of pregnancy was <1%, despite missing two active pills per cycle [26]. No pregnancies were reported during the study. However, contraceptive efficacy was not directly evaluated in this study, and the number of exposed cycles was not recorded. Still it can be assumed that based on the drug’s mode of action only, efficacy in the adolescent population is expected to be equivalent to that in the population aged 16 and above. [21].

The use of estetrol/drospirenone in adolescents resulted in cycle control and a bleeding pattern comparable to the predictable profile observed in the adult population [27], primarily due to a reduction in unscheduled bleeding. In pooled adult phase 3 studies, the incidence of unscheduled bleeding and/or spotting declined from 27.1% in Cycle 1 to 20.6% in Cycle 2, and to ≤17.5% from Cycle 5 onwards. Among those who experienced unscheduled bleeding and/or spotting, the median number of days remained stable throughout the study, ranging from three to four days. Scheduled bleeding or spotting was reported in 87.2% to 90.4% of participants per cycle, with a median duration of four to five days. Absence of scheduled bleeding/spotting varied according to the body mass index of the participants (7.6–21.6% per cycle) [27].

Up to 90% of adolescents experience menstrual discomfort, primarily due to dysmenorrhea [5,28] which often leads to missed school and other activities. Severe cases of dysmenorrhea may impact quality of life and increase the risk for depression and anxiety [29,30]. While non-steroidal anti-inflammatory drugs (NSAIDs) are the first-line treatment, COCs are also effective in reducing the symptoms of primary dysmenorrhea [31,32] and are regularly prescribed when NSAID treatment is not sufficient [5]. In our study, where most participants reported a history of dysmenorrhea, estetrol/drospirenone treatment reduced the pain score related to dysmenorrhea by >36%, and this reduction was even more pronounced (>44%) in those with high baseline symptoms. This positive trend was further supported by a decrease of 32% in the use of pain medication. These findings are consistent with results in adults, where estetrol/drospirenone demonstrated superior efficacy compared to placebo in reducing VAS scores for pelvic pain and dysmenorrhea symptoms after four cycles in Japanese patients with primary and secondary dysmenorrhea [33,34].

This is one of the few studies that have evaluated the safety of a COC in adolescents. While paediatric studies are essential for evaluating new drugs, we found that recruiting participants was challenging due to various legal, cultural, and ethical barriers. Parental consent requirements, even from one parent, hindered recruitment, while in real life, this age group can often access contraception without parental approval. Additionally, cultural norms made recruiting adolescents interested in contraception complex, leading some sites to recruit participants seeking prescriptions for therapeutic purposes (such as dysmenorrhea) instead.

Several methodological limitations should be acknowledged. In this study, we enrolled participants between 12 and 17 years of age, primarily first-time COC users, the majority with a history of dysmenorrhea. Their characteristics may differ from the real-world adolescent population seeking COCs for contraceptive or therapeutic use, as we intentionally included young adolescents in our study, to extend the evaluation of safety also to this younger population. Because this was an open-label, single-arm study, the influence of placebo or expectation effects, particularly on subjective outcomes such as dysmenorrhea, cannot be excluded. The relatively small sample size and attrition over treatment cycles reduced the number of evaluable participants and, consequently, the precision of later-cycle analysis in particular for dysmenorrhea, and therefore we presented data based on last observation carried forward. While the six-month duration and sample size of 105 participants were sufficient to meet regulatory requirements for assessing the safety of estetrol/drospirenone in adolescents, the short duration limits the assessment of the bleeding profile, especially given the potential for menstrual bleeding-related problems in this population. We acknowledge that limitations in participant compliance with daily diary entries and pill use reporting may have impacted data completeness and the interpretation of some secondary outcomes. To ensure data accuracy, we established clear criteria for handling missing data (Appendix B Table A2). Although there was a slight decrease in the number of evaluable participants from Cycles 1 to 5, the sample size remained sufficient to allow a consistent analysis of bleeding patterns. The most substantial reduction occurred in Cycle 6, which included only 39 evaluable cycles. This time point, however, was not eligible for bleeding pattern analysis, as it extended three days beyond the study end date. Due to the study design and sample size, dysmenorrhea was assessed only by VAS scores and rescue medication use, which may not fully capture the complexity of the symptoms. Averaging of the three highest VAS scores to characterise peak pain, may slightly overestimate baseline pain and the magnitude of change during treatment. Thus, clinicians should evaluate the pill’s impact individually based on each user’s experience. Although some switchers were included, their prior pill use likely had minimal effect due to their small number and a one-month washout period before baseline evaluation. Finally, most participants were White and from Northern or Eastern Europe, limiting generalisability to more diverse populations. However, as a regulatory study under the EMA-approved Pediatric Investigation Plan, it was designed to provide safety and tolerability data supporting estetrol/drospirenone use in adolescents, offering important evidence for European clinical practice where COCs are widely used for both contraception and menstrual disorders.

Overall, this study demonstrated that the use of estetrol/drospirenone in adolescents was not associated with safety concerns, was well tolerated, resulted in a clear and stable cyclic bleeding pattern aligned with the profile observed in adult studies, and reduced pain associated with dysmenorrhea, making it a promising option for adolescents. These findings should be interpreted with the limitations of the study design in mind and in consideration of the individual needs of each user. Further studies are needed to confirm and expand these results.

## Figures and Tables

**Table 1:** Demographic data and baseline characteristics of participants in a six-month paediatric study with estetrol/drospirenone (N = 105).

Variable	Estetrol/Drospirenone 15 mg/3 mg (N = 105)
**Age** (years)	
Mean (SD)	15.2 (1.21)
Median (min, max)	15.6 (12, 17)
**Body mass index** (kg/m^2^)	
Mean (SD)	21.2 (2.98)
Median (min, max)	20.7 (16, 31)
**Race**	
White	98 (93.3)
Not collected	4 (3.8)
Asian	1 (1.0)
Black or African American	2 (1.9)
**Ethnicity**	
Not Hispanic or Latino	95 (90.5)
Not collected	10 (9.5)
**Gynaecological history**	
Gravidity = 0	105 (100.0)
Parity = 0	105 (100.0)
History of dysmenorrhea	88 (83.8)
Contraceptive use status—starter	96 (91.4)
Contraceptive use status—switcher ^a^	9 (8.6)
**Pre-treatment menstrual cycle characteristics ^b^**	
Cycle length in days (n = 100)	
Mean (SD)	30 (9.78)
Median (min, max) ^c^	30 (2, 46)
**Discontinuation ^d^** (%)	15 (14.3)

Safety population. Data are presented as the number (percentage) of participants unless stated otherwise. ^a^ Past contraceptive use within two months before screening (switchers). ^b^ Pre-treatment cycle is defined as the last menstrual cycle before the start of estetrol/drospirenone use. ^c^ Median range due to the protocol deviation. ^d^ Reasons for discontinuation were withdrawal of consent (n = 8; 7.6%), lost to follow-up (n = 2; 1.9%), other reasons (n = 2; 1.9%), adverse event (n = 1; 1.0%), physician decision (n = 1; 1.0%) and protocol deviation (admission criteria violation, n = 1; 1%). SD: standard deviation.

**Table 2:** Summary of Treatment-Emergent Adverse Events (Safety population) reported in a six-month paediatric study with estetrol/drospirenone (N = 105).

	Estetrol/Drospirenone 15 mg/3 mg (N = 105)	
Adverse Events ^a^	Number of Subjects n (%)	Number of Events n
**Any adverse events**	54 (51.4)	113
**Adverse events reported in ≥2% of participants**		
Headache	13 (12.4)	17
Nasopharyngitis	10 (9.5)	14
Nausea	5 (4.8)	5
Abdominal pain	4 (3.8)	6
COVID-19	4 (3.8)	4
Vomiting	3 (2.9)	4
Intermenstrual bleeding	3 (2.9)	3
Menstruation delayed	3 (2.9)	3
Lipase increased	3 (2.9)	3
**Serious adverse events**	0	0
**Drug-related adverse events ^b^**	12 (11.4)	15
Dysmenorrhea	2 (1.9)	2
Nausea	2 (1.9)	2
Abdominal pain	1 (1.0)	1
Abnormal uterine bleeding	1 (1.0)	1
Acne	1 (1.0)	1
Breast tenderness	1 (1.0)	1
Depressed mood	1 (1.0)	1
Dizziness	1 (1.0)	2
Heavy menstrual bleeding	1 (1.0)	1
Menstruation delayed	1 (1.0)	1
Menstruation irregular	1 (1.0)	2
**Adverse events leading to discontinuation ^c^**	1 (1.0)	2
Dysmenorrhea	1 (1.0)	1
Heavy menstrual bleeding	1 (1.0)	1

The adverse events shown are treatment-emergent adverse events that began after the start of study treatment. ^a^ Medical term coded according to Medical Dictionary for Regulatory Activities version 24.1. ^b^ Relation to the study drug was established by the site investigator. ^c^ One subject discontinued treatment due to dysmenorrhea and heavy menstrual bleeding related to the study drug, having taken only four doses. One additional participant discontinued the study with a report of a non-drug-related adverse event (amenorrhea), with the primary reason for discontinuation being “protocol deviation” (pre-treatment cycle > 45 days). N: number of participants in safety set, n: number of participants with adverse event, % based on N.

## Data Availability

The data presented in this study are available on request from the corresponding author due to legal reasons.

## Abbreviations

AE	Adverse event
COC	Combined oral contraceptive
COVID-19	Coronavirus disease 2019
CfB	Changes from baseline
DRSP	Drospirenone
ECG	Electrocardiogram
EoT	End of treatment
EMA	European Medicines Agency
E4	Estetrol
MedDRA	Medical Dictionary for Regulatory Activities
NSAID	Non-steroidal anti-inflammatory drug
PIP	Paediatric Investigation Plan
SD	Standard deviation
TEAE	Treatment-emergent adverse event
VAS	Visual analogue scale

## Appendix A

**Table A1:** Bleeding and Spotting Definitions used in a six-month pediatric study with estetrol/drospirenone (N = 105).

Term	Definition
Vaginal bleeding	Evidence of vaginal blood loss that requires the use of sanitary protection with a tampon, menstrual cup, pad or pantyliner.
Vaginal spotting	Evidence of minimal vaginal blood loss that does not require new use of sanitary protection, including pantyliners.
Episode of vaginal bleeding/vaginal spotting	Bleeding/spotting days bounded on either end by 2 days of no bleeding or spotting.
Scheduled vaginal bleeding/vaginal spotting episode	The episode has a start date within Day 25 of the cycle and Day 3 of the next cycle, or starts before Day 25 and stops before Day 3 of the next cycle. A scheduled vaginal bleeding/vaginal spotting episode has therefore: - A start date and stop date in the scheduled bleeding interval (Day 25–Day 3 next cycle) OR - A start date before Day 25 and stop date before Day 3 (Early withdrawal Bleeding/Spotting) OR - A start date in the scheduled bleeding interval (Day 25–Day 3 next cycle) and continuing into next cycle (Continued withdrawal Bleeding/Spotting)
Unscheduled vaginal bleeding/vaginal spotting episode	The episode is defined as unscheduled if it does not meet the definition for scheduled vaginal bleeding/spotting.

## Appendix B

**Table A2:** Missing data curation used in a six-month pediatric study with estetrol/drospirenone (N = 105).

Endpoint Evaluation	Definition
Missing bleeding data	The primary bleeding analysis will include all subjects in the respective analysis population who have 1 evaluable cycle. Cycles will be considered evaluable if no more than two consecutive days with missing bleeding information occurred within the cycle. One or two consecutive days with missing bleeding information will be imputed with the bleeding information from the previous day.
Missing concomitant medications dates	For determining whether a medication is taken during the study treatment period, the following rules for missing and partial dates will be applied: If the end date is before the first dose of study medication, then the medication is a prior, regardless of whether or not the start date is missing or partially missing. If the end date is on or after the date of the first dose of study medication, then the following rules will be applied for missing or partially missing start dates: o If the start date is completely missing, then the medication will be assumed to be concomitant. - If only the year of the start date is recorded and the year is either before or the same year as the end of the safety study treatment period, then the medication will be assumed to be concomitant. - If only the year of the start date is recorded and the year is after the year of the end of the study treatment period, it will be considered to be a post-study treatment medication. - If only the year and the month of the start date is recorded and the month specified is either before or the same month as the end of the study treatment period assuming the same year, then the medication will be assumed to be concomitant. - If only the year and the month of the start date is recorded and the month specified is after the month of the end of the study treatment period assuming the same year, it will be considered to be a post-study treatment medication.
Missing VAS score data for the Cycle 6	For subjects with Cycle 6 missing or not being evaluable the Cycle 6 score will be obtained from the last evaluable cycle (Last Observation Carried Forward imputation). The Last Cycle score will be obtained based on the data from day 1 of the last cycle to the last day of that cycle—Last Cycle score.

VAS: Visual analogue score.

## Appendix D

**Table A3:** Use of pain medication for dysmenorrhea in a six-month pediatric study with estetrol/drospirenone (N = 105).

Timepoint	N	Participants with ≥1 Use of Pain Medication n (%)	Number of Days with Pain Medication, Mean (SD)
Baseline ^a^	81	53 (63.9)	2.1 (5.0)
Cycle 1	71	40 (56.3)	2.1 (3.6)
Cycle 3	68	26 (38.2)	0.9 (1.7)
Cycle 6	38	12 (31.6)	0.6 (1.0)

^a^ Recorded in pre-treatment cycle. N: number of evaluable participants in each cycle; SD: standard deviation.

## Appendix E

**Table A4:** Absolute values at baseline and cycles 1, 3 and 6 of average three highest VAS score assessments for dysmenorrhea.

Cycle (N)	Mean (SD)	Median (Min, Max)	CfB Mean (SD)	CfB Median (Min, Max)	Percent CfB Mean (SD)	Percent CfB Median (Min, Max)
Baseline (83)	4.59 (2.477)	5.0 (0.0, 9.7)	-	-	-	-
Cycle 1 (65)	4.88 (5.685)	5.0 (0.0, 10.0)	−0.05 (2.519)	0.3 (−6.3, 5.0)	16.18 (87.385)	6.7 (−100.0, 400.0)
Cycle 3 (61)	3.18 (2.475)	3.0 (0.0, 10.0)	−1.90 (2.750)	−1.7 (−7.7, 4.3)	−20.19 (112.250)	−36.4 (−100.0, 700.0)
Cycle 6 (35)	3.51 (2.618)	3.7 (0.0, 9.0)	−1.30 (3.439)	−1.3 (−8.7, 7.3)	36.76 (323.456)	−34.8 (−100.0, 1800.0)
Last Cycle * (72)	3.38 (2.595)	3.5 (0.0, 9.0)	−1.63 (3.047)	−1.3 (−8.7, 7.3)	2.42 (230.778)	−33.3 (−100.0, 1800.0)

N: number of evaluable participants in each cycle; CfB: changes from baseline; SD: standard deviation; min: minimum; max: maximum. Baseline count is determined for the values recorded in the pre-treatment cycle. Only evaluable cycles 1, 3 and 6 are used. * Last Cycles include Cycle 6 if available or the assessment from the last available evaluable cycle 1, 3 or 6 otherwise (Last Observation Carried Forward imputation at Cycle 6).
